# Prediction of poor outcomes six months following total knee arthroplasty in patients awaiting surgery

**DOI:** 10.1186/1471-2474-15-299

**Published:** 2014-09-08

**Authors:** Eugen Lungu, François Desmeules, Clermont E Dionne, Étienne L Belzile, Pascal-André Vendittoli

**Affiliations:** Department of Biomedical Sciences, Faculty of Medicine, University of Montreal, Montreal, QC Canada; Orthopaedic Clinical Research Unit, Maisonneuve-Rosemont Hospital University of Montreal affiliated Research Center, CP 6128 Succursale Centre-Ville, Montréal, H3C 3 J7 Quebec, Canada; School of Rehabilitation, Faculty of Medicine, University of Montreal, Montreal, QC Canada; URESP, Centre hospitalier universitaire (CHU) de Québec, Quebec, QC Canada; Department of Rehabilitation, Faculty of Medicine, Laval University, Quebec, QC Canada; Centre hospitalier universitaire (CHU) de Québec, Quebec, QC Canada; Department of Surgery, Faculty of Medicine, University of Montreal, Montreal, QC Canada

**Keywords:** Total knee arthroplasty, Osteoarthritis, Prediction rule, Determinants

## Abstract

**Background:**

Identification of patients experiencing poor outcomes following total knee arthroplasty (TKA) before the intervention could allow better case selection, patient preparation and, likely, improved outcomes. The objective was to develop a preliminary prediction rule (PR) to identify patients enrolled on surgical wait lists who are at the greatest risk of poor outcomes 6 months after TKA.

**Methods:**

141 patients scheduled for TKA were recruited prospectively from the wait lists of 3 hospitals in Quebec City, Canada. Knee pain, stiffness and function were measured 6 months after TKA with the Western Ontario and McMaster Osteoarthritis Index (WOMAC) and participants in the lowest quintile for the WOMAC total score were considered to have a poor outcome. Several variables measured at enrolment on the wait lists (baseline) were considered potential predictors: demographic, socioeconomic, psychosocial, and clinical factors including pain, stiffness and functional status measured with the WOMAC. The prediction rule was built with recursive partitioning.

**Results:**

The best prediction was provided by 5 items of the baseline WOMAC. The rule had a sensitivity of 82.1% (95% CI: 66.7-95.8), a specificity of 71.7% (95% CI: 62.8-79.8), a positive predictive value of 41.8% (95% CI: 29.7-55.0), a negative predictive value of 94.2% (95% CI: 87.1-97.5) and positive and negative likelihood ratios of 2.9 (95% CI: 1.8-4.7) and 0.3 (95% CI: 0.1-0.6) respectively.

**Conclusions:**

The developed PR is a promising tool to identify patients at risk of worse outcomes 6 months after TKA as it could help improve the management of these patients. Further validation of this rule is however warranted before clinical use.

**Electronic supplementary material:**

The online version of this article (doi:10.1186/1471-2474-15-299) contains supplementary material, which is available to authorized users.

## Background

Total knee arthroplasty (TKA) surgery is widely regarded as the treatment of choice for patients suffering from knee osteoarthritis (OA) once the options for conservative treatment have been exhausted [1]. TKA is the second most popular type of orthopaedic surgery and projective data suggests a six-fold increase in the number of primary TKAs in the following decades in North America [2]. TKA is an effective procedure and the majority of patients will show important improvements in pain, disabilities and health-related quality of life [3]. However, a growing body of evidence suggests that 10-30% of patients undergoing TKA have very poor or no improvement following surgery [4–7] Several factors that are associated to such negative outcomes have been identified; inappropriate expectations, contralateral knee pain, higher psychological distress, high body mass index, use of a walking aid, advanced age, female gender, lower OA grade and thyroid disease have all been found to be significantly associated to worse physical function following TKA [4, 8–22]. Nonetheless, these findings are often not consistent across studies and the exact strength of the associations between these factors and the outcomes remain elusive. It thus remains a challenge to identify which TKA candidates will likely do well, or do poorly following TKA [8].

The fact that the surgery might not be successful for many patients prompts the necessity of identifying those who are at the greatest risk of having poor outcomes following TKA. Their identification could orient both clinicians and patients regarding the decision of undertaking the procedure [9]. Moreover, medical or rehabilitation interventions could be initiated preoperatively or postoperatively [10]. Successful identification of patients at risk of adverse outcomes after TKA could not only benefit patients, but also clinicians and policy makers in more efficiently allocating necessary healthcare resources required by the condition of these patients [10, 23].

By developing an accurate and easy-to-use prediction tool, better case management of patients enrolled on a wait list for TKA could be achieved. To our knowledge, no such tool has ever been developed for this population. Few clinical predictive rules have been built and validated to allow for better case management of other types of musculoskeletal complaints. The Ottawa Ankle and Knee Rules are used in order to identify the need for roentgenographic investigation following acute ankle and knee injuries respectively [24, 25]. The Cassandra Rule has been developed to identify patients with non-specific back pain that are most likely to develop or sustain long-term functional limitations [26]. Therefore, the objective of the present study was to develop a prediction rule (PR) that would allow a better identification of patients at the greatest risk of poor outcome six months after TKA upon enrolment on a wait list for surgery.

## Methods

### Study design

This study employed a prospective longitudinal design with repeated measures. It was part of a broader study targeted at measuring the effects of wait time on patients undergoing TKA [11, 27]. It adheres to the Strengthening the Reporting of Observational Studies in Epidemiology (STROBE) guidelines for observational cohort studies (see Additional file 1).

### Settings

From 02/2006 to 09/2007, patients newly included on the waiting lists of the departments of orthopaedic surgery of three teaching hospitals in Quebec City, Canada (CHUL, HSFA and HDQ) were recruited. Follow-up of participants ended in 09/2010 because of the extensive wait times in the participating hospitals. All seven orthopaedic surgeons performing TKA in these three hospitals collaborated in the study.

### Participants

Every week, patients newly enrolled on the surgical wait lists of the three hospitals were contacted by a research nurse by phone. Eligible subjects had to meet the following inclusion criteria: (1) age ≥ 40 years old; (2) scheduled for primary unilateral TKA; 3) understands, reads and speaks French. Patients were excluded if they were suffering from a severe cardiac condition, a severe degenerative disease (other than OA) such as Parkinson’s disease, Alzheimer’s disease, any type of dystrophies or other type of sclerosis with the potential to interfere with patient recovery following TKA or any severe mental disorder (severe depression, bipolar disorder, schizophrenia or dementia) that could interfere with the ability to answer the protocol questionnaires. Subjects with a previous joint arthroplasty (hip or knee) were also excluded. Those who suffered a major trauma to the knee in the previous year or underwent surgery urgently within 30 days of registration on the waiting list were further excluded.

### Data collection

Data were collected via a review of the patients’ medical files and structured 45 minutes phone interviews conducted by three trained interviewers. The interviews were performed a few days after enrolment on the wait lists (mean ± SD: 12.6 ± 4.7 days) and six months after the TKA (mean ± SD: 188.7 ± 5.4 days). Patients were also interviewed before surgery; these results have been reported previously [11].

### Dependent variables

Pain, stiffness and function at enrolment and six months after surgery were measured with the Western Ontario and McMaster Osteoarthritis Index (WOMAC), a 24-question tool [28]. The WOMAC has been found to have very good reliability, convergent construct validity and responsiveness, and has been used extensively with similar populations [29–31]. The WOMAC score was transformed in order to obtain a score that varied from 0 to 100, 0 indicating no pain, no functional limitations nor knee joint stiffness. As there is no universal agreement on what is considered poor outcome following TKA surgery, it was defined as the last quintile of the six-month postoperative WOMAC score (i.e. WOMAC score > 40.4); a satisfactory outcome was defined by a WOMAC score in the first four other quintiles of the distribution (i.e. score ≤ 40.4).

### Independent variables

Independent variables collected to be considered as potential predictors in the final predictive model included known important determinants of TKA outcomes reported in the literature [4, 8–22] Variables were measured at enrolment on the wait list and 6 months after TKA.

### Potential predictors at enrolment on surgical wait list

Initial diagnosis, anthropometric data and comorbidities were recorded from the subjects’ medical files. The burden of comorbidities was assessed using the Cumulative Illness Rating Scale [32]. At the initial interview, questions drawn from the questionnaire of the 1998 Quebec Health Survey were used to measure formal education, employment status, and household income. Social support was also measured with questions from the Quebec Health Survey [33]. Marital status, household living status, and clinical variables such as duration of disease symptoms were also noted during the initial interview. Psychological distress was recorded with a modified version of the Psychological Symptom Index (PSI). The modified PSI includes 13 questions that measure depression and anxiety during the past week (range: 0–42) [34]. We also considered individual questions from validated questionnaires (i.e.: social support tool, PSI and WOMAC) to build the rule. This was done in an effort to simplify the number of items to include in the final PR.

### Other variables

Several surgical variables such as type of implant, bearing type, implant fixation, patella resurfacing and the number and type of in-hospital complications (wound infection, dislocation, knee ankylosis and manipulation, cardiovascular/pulmonary/circulatory complications, peripheral/central nervous system involvement, urinary infection, acute confusion, tendon and ligament rupture, blood transfusion) following TKA were recorded by reviewing the subjects’ medical files. The same procedure was used to document hospital length of stay and discharge to a rehabilitation or recovery facility. The pre-surgery wait times were calculated from the data extracted from the wait list database of each hospital. Six months following the surgery, patients were asked about walking aid usage and the number of community physiotherapy treatment hours received since discharge from the hospital.

### Statistical analysis

Less than 2% of the data of the WOMAC questionnaire was missing, and it was handled according to the recommendations of the tool’s guidelines [28]. Recursive partitioning analyses were used to build the PR. One of the most effective algorithm is Classification and Regression Trees [35]. It relies on considering all combinations of the predictors in order to maximize homogeneity within nodes. The Gini heterogeneity coefficient was used as a criterion to build the models [36]. Since the sample size was relatively small, we used all data in the training set. An automatic approach was first used to build PRs. Then, a set of eligible candidate predictors was created by manual adjustment based on statistical, clinical and ease of use considerations. For each resulting PR, sensitivity, specificity, Area Under the receiver operating characteristics – ROC – Curve (AUC), predictive value of positive and negative tests, as well as positive and negative likelihood ratios were calculated with their 95% confidence intervals [37]. The simplest rule demonstrating the highest sensitivity with acceptable level of specificity was selected as the final tool. The accuracy of the proposed model using 1,000 bootstrap resamples was then calculated for internal validation [38]. All analyses were carried out using SPSS Answer Tree 3.1 (SPSS Inc., Chicago) and SAS statistical suite software version 9.2 (SAS Institute Inc., Cary, NC, U.S.A.).

### Ethics

All participants signed an informed consent form. The study was approved annually by the Research Ethics Boards of all three hospitals (CHUL, HSFA and HSFA).

## Results

### Participants

**Figure 1:**
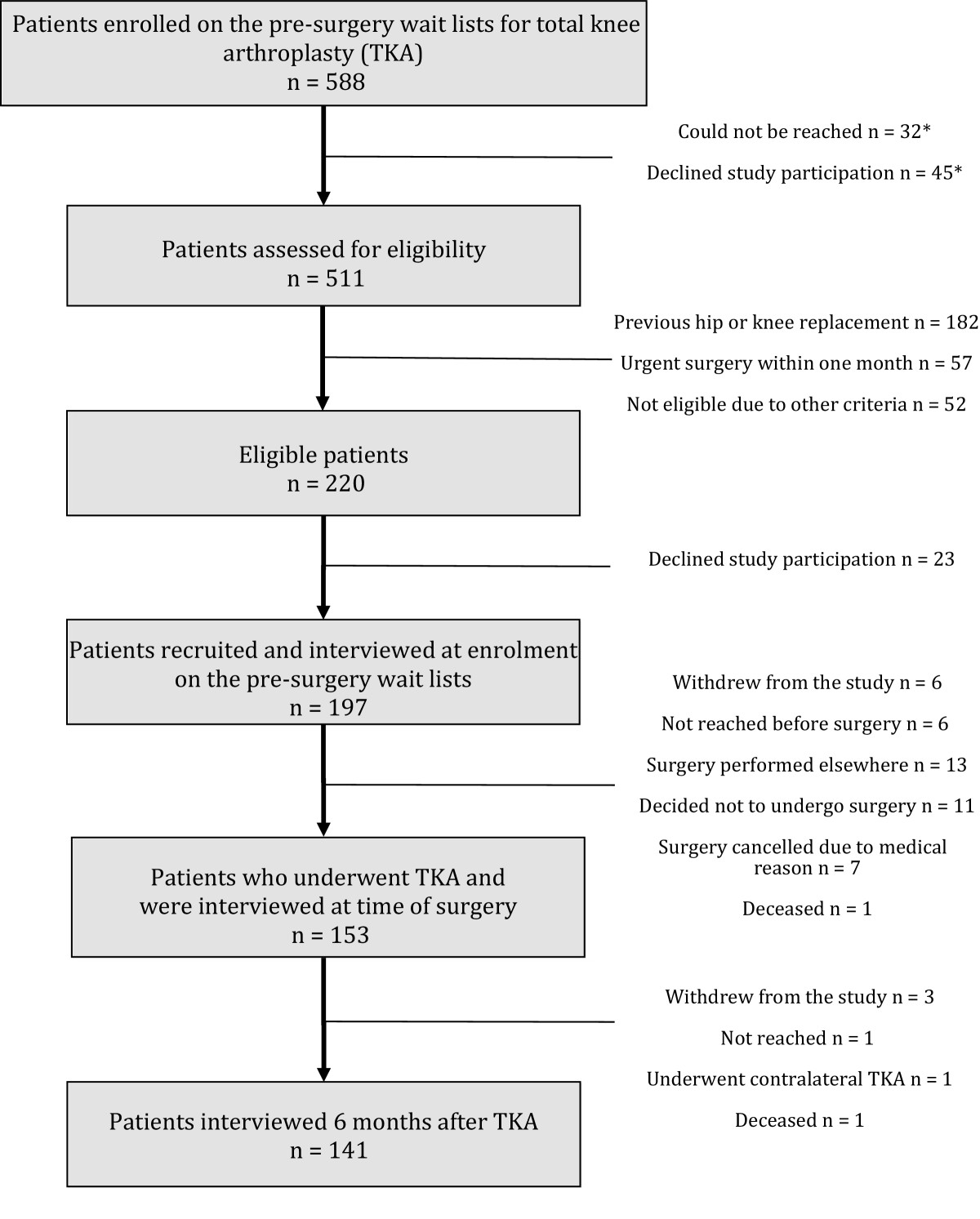
**Flowchart of patients’ recruitment.** *Eligibility status unknown (considered in calculation of participation proportion). TKA, total knee arthroplasty.

Figure 1 shows the flow of subjects through the duration of the study. A total of 588 patients enrolled on the wait list of the three hospitals. Thirty-two patients could not be reached within 3 weeks of inclusion on the wait list and 45 declined participation. Out of 511 patients whose eligibility was assessed, 220 patients met the eligibility criteria. Following 23 further declinations, 197 patients were interviewed at the time of enrolment on the wait list. A further six patients withdrew from the study. Six others could not be reached before surgery. Thirteen had surgery performed in a different institution. Eleven decided not to undergo surgery. The surgeries of 7 patients were cancelled due to medical reasons. One patient passed away while waiting for TKA and one after the surgery was performed (both deaths unrelated to TKA). Therefore, 153 patients underwent TKA. Of these, three withdrew from the study, one could not be reached six months after the surgery and seven underwent contralateral knee arthroplasty within six months. A total of 141 patients were thus interviewed six months after TKA. The overall eligibility proportion was calculated as (220/511) = 0.43; the participation proportion was calculated as (197/(220 + ((45 + 32) × 0.43))) = 77.8%, and the follow-up proportion was calculated as (141/(197 – 44)) = 92.2%.

Participants had a mean age of 66 (SD: ± 9.5) years. The majority of patients were women (66%) and suffered from contralateral knee pain (72%). The mean wait time of the participants was 184 (SD: ± 120.8) days and median wait time was 148 days (range: 32–692). The majority of TKA implants were postero-stabilized (82%) and cemented (96%). Mean hospital length of stay was 7.5 days (SD: ± 3.0) (Table 1).

**Table 1:** **Selected characteristics of the participants who underwent primary unilateral total knee arthroplasty surgery n = 141**

Variables considered for PR development	n (%)	Mean (SD)	Other collected variables	n (%)	Mean (SD)
Demographics			Pre-surgery wait		
Age (years)		66 (9.5)	Time between enrolment on wait list and surgery (days)^*^		184 (120.8)
Female	93 (66)		Categories of wait time		
Marital status			≤3 months	30 (21)	62.5 (16.9)
Single, separated, divorced or widowed	51 (36)		>3-6 months	53 (38)	130.2 (28.7)
Married or common law	90 (64)		>6-9 months	31 (22)	216.8 (25.1)
Living alone	34 (24)		>9 months	27 (19)	386.3 (56.7)
Socioeconomic characteristics			Surgery postponed for personal reasons	9 (6)	
Educational level (part or complete)			Surgical characteristics		
High school or less	79 (56)		Implant type		
College or University	62 (44)		Postero-stabilized	115 (82)	
Employment status			Cruciate retaining	26 (18)	
Unemployed or retired	108 (77)		Implant fixation		
Employed	33 (23)		Cementless	4 (3)	
Household income^**^			Hybrid	2 (1)	
< $30 000 / year	48 (34)		Cemented	135 (96)	
$30 000 - $59 999 / year	43 (31)		Implant bearing type		
≥ $60 000 / year	34 (24)		Mobile	4 (3)	
Missing data	16 (11)		Fixed	137 (97)	
Psychosocial characteristic*s*			Patella resurfacing	132 (93)	
Psychological distress (/42)		7.2 (7.0)	In-hospital complications^‡^		
Social support°			0	109 (77)	
Low	67 (48)		1	23 (16)	
High	74 (52)		≥2	10 (7)	
Clinical characteristics			Health services utilization		
Diagnosis			Hospital length of stay (days)		7.5 (3.0)
Osteoarthritis	136 (96)		Discharged directly home	123 (87)	
Rheumatoid arthritis	5 (4)		Post-surgery community physiotherapy (hours)		14.7 (18.7)
BMI^¬^ (kg/m^2^)		31.2 (6.2)			
Comorbidities (/56)		6.5 (2.2)			
Duration of knee symptoms before enrolment^†^ (years)		7.9 (8.1)			
Contralateral knee pain^§^	101 (72)				
Use of a walking aid					
At enrolment on wait list	55 (39)				

SD: standard deviation.

^*^Median (range): 148 days (32–692).

^**^n = 125 – CND $.

°Social support was dichotomized around the median score: Low (≤80) and High (>80).

^¬^Body mass index.

^†^n = 138.

^§^WOMAC pain score at enrolment on pre-surgery wait list dichotomized into presence or absence of contralateral knee pain.

^‡^In-hospital complications including: wound infection, dislocation, knee ankylosis and manipulation, cardiovascular/pulmonary/circulatory complications, peripheral/central nervous system involvement, urinary infection, acute confusion, tendon and ligament rupture or blood transfusion.

Six months following TKA, participants showed a significant improvement in terms of both pain (-30.6, SD: ± 21.8, 95% CI -26.9 to -34.2), stiffness (-26.0, SD: ± 20.4, 95% CI -21.2 to -30.8), and function mean scores (-25.4, SD: ± 20.5, 95% CI - 22.0 to -28.8), as well as in overall WOMAC mean score (-27.3, SD: ± 15.8, 95% CI -23.6 to -31.0) (Table 2).

**Table 2:** **Overall changes in WOMAC scores of the participants between enrolment on the pre-surgery waiting lists and 6 months after TKA (n = 141)**

	Mean score at enrolment^†^(SD)	Mean score 6 months after TKA^†^(SD)	Change in score^‡^(SD)	95% CI	Comparison between time points***(p*** value)
**WOMAC**					
**Pain**	53.1 (17.9)	22.5 (17.1)	- 30.6 (21.8)	- 26.9 to - 34.2	<0.001*
**Stiffness**	59.3 (19.7)	33.3 (21.1)	- 26.0 (20.4)	- 21.2 to - 30.8	<0.001*
**Function**	53.5 (14.3)	28.1 (17.9)	- 25.4 (20.5)	- 22.0 to - 28.8	<0.001*
**Total score**	55.3 (15.2)	28.0 (16.3)	- 27.3 (15.8)	- 23.6 to - 31.0	<0.001*

SD: standard deviation.

CI: confidence interval.

^**†**^Scores presented as standardised scores. Lower scores sign a better condition.

^‡^Negative changes in score sign an improvement of the condition.

*p < 0.05.

### Final prediction rule

**Figure 2:**
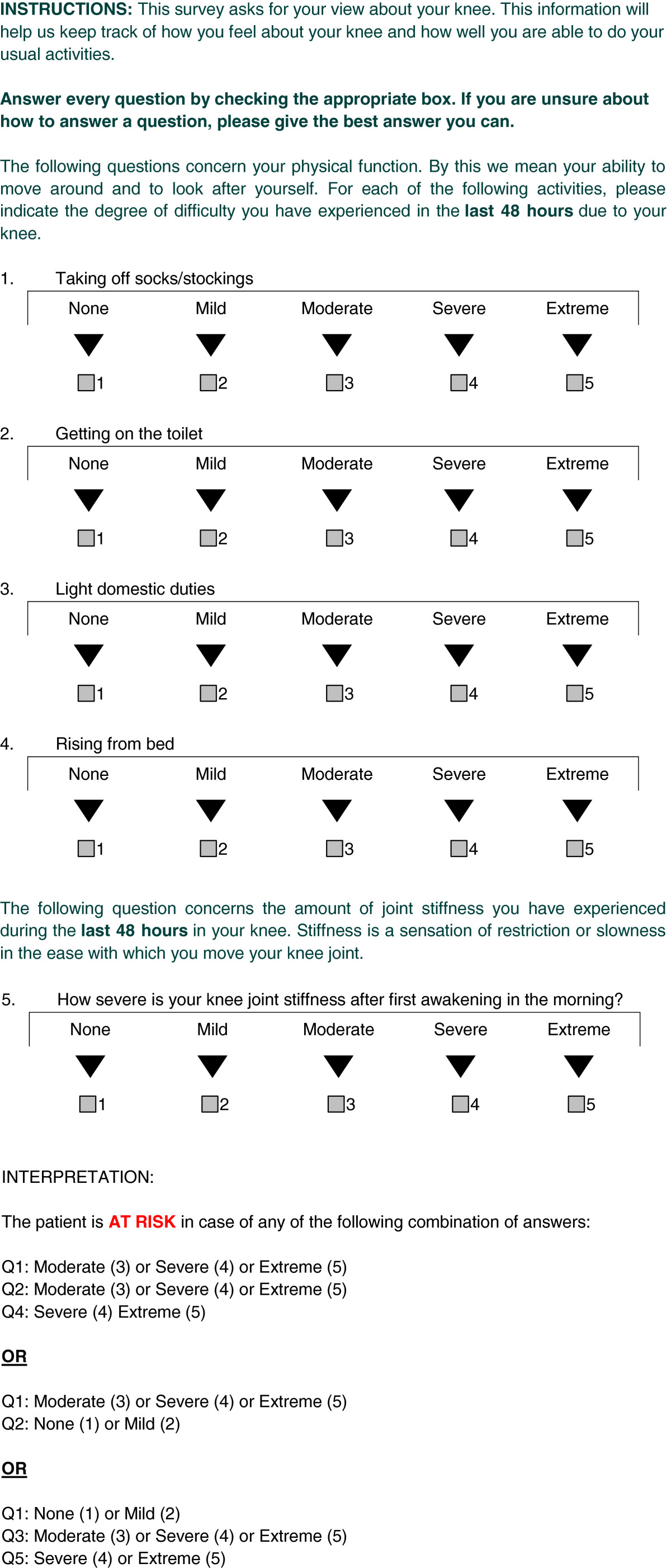
**Prediction algorithm to identify patients at risk of poor outcome following TKA.**

**Figure 3:**
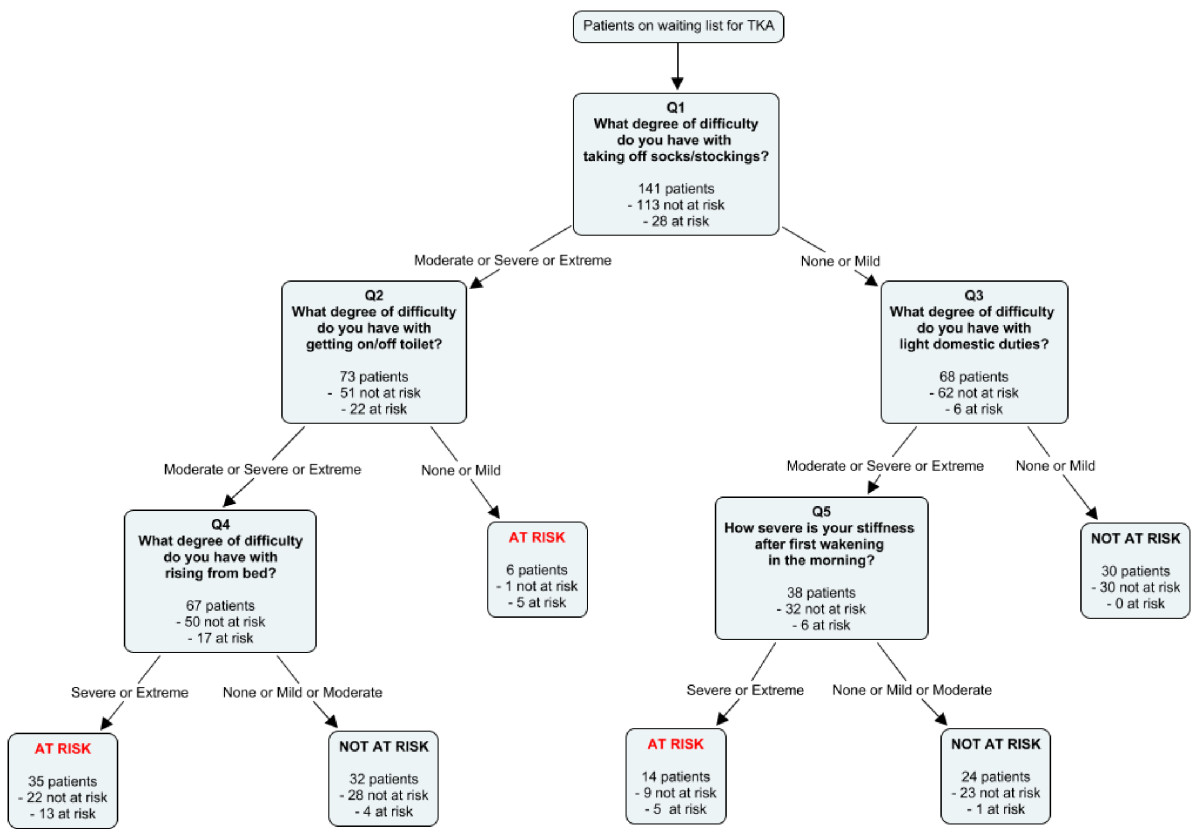
**Graphical representation of the PR and its interpretation.**

Overall, out of the 141 participants who completed this study, 28 (20%, corresponding to the first quintile of the distribution) scored ≥ 40.4% (total score) on the WOMAC questionnaire, thus being identified as patients with the worst outcomes. From all potential predictors measured at enrolment, the final PR included the answers to 5 questions drawn from the WOMAC at baseline: preoperative difficulty of taking off socks, getting on/off toilet, performing light domestic duties and rising from bed as well as degree of morning stiffness after the first wakening (Figure 2). The patients answered these questions in a sequential manner by attributing a degree of difficulty (none, mild, moderate, severe or extreme) to the items described in the questions. Depending on the pattern of their answers, the patients could be classified as either at risk or not at risk for poor outcomes (Figure 3).

The final PR correctly identified 23 of the 28 patients with the worst outcomes and 81 of the 113 patients with the best outcomes (Table 3). Therefore, it had a sensitivity of 82.1% (95% CI 64.4 to 92.1), a specificity of 71.7% (95% CI 62.8 to 79.2) and a positive likelihood ratio of 2.901 (95% CI 2.064 to 4.077).

**Table 3:** **Two by two table of predicted versus actual outcomes of the final PR**

	Actual outcome	
**Predicted outcome**	**AT RISK**	**NOT AT RISK**
	**(Post-operative WOMAC > 40.4)**	**(Postoperative WOMAC ≤ 40.4)**
**AT RISK**	23	32
**NOT AT RISK**	5	81
*TOTAL*	*28*	*113*

Presented in the appendix are other prediction models developed that were also considered (see Additional file 2).

### Internal validation

The accuracy of the rule was confirmed using 1,000 bootstrap resamples For each and every measure of predictive validity, the estimate obtained with the bootstrap was very close to the original estimate (Table 4).

**Table 4:** **Validity measures of the predictive rule**

Measure	Estimates in training sample	Estimates with 1,000 bootstrap resamples
***Sensitivity% (95% CI)***	82.1 (64.4-92.1)	82.1 (66.7-95.8*)
***Specificity% (95% CI)***	71.7 (62.8-79.2)	71.7 (62.8-79.8*)
***Positive predictive value% (95% CI)***	41.8 (29.7-55.0)	41.8 (29.1-55.8*)
***Negative predictive value% (95% CI)***	94.2 (87.1-97.5)	94.2 (88.8-98.8*)
***Positive likelihood ratio (95% CI)***	2.90 (2.06-4.08)	2.90 (1.81-4.74*)
***Negative likelihood ratio (95% CI)***	0.25 (0.11-0.57)	0.25 (0.11-0.58*)
***Area under ROC curve (95% CI)***	0.77 (0.69-0.85)	0.77 (0.69-0.85*)

• *95% asymptotic confidence intervals.

Sensitivity: number of participants classified at risk both by the PR and the post-operative WOMAC score divided by all participants classified at risk by the post-operative WOMAC score (actual outcome).

Specificity: number of participants classified not at risk by the PR and the post-operative WOMAC score divided by all participants classified not at risk by the post-operative WOMAC score (actual outcome).

Positive predictive value: number of participants classified at risk by the PR and the post-operative WOMAC score divided by all participants classified at risk by the PR (predicted outcome).

Negative predictive value: number of participants classified not at risk by the PR and the post-operative WOMAC score divided by all participants classified not at risk by the PR (predicted outcome).

Positive likelihood ratio: sensitivity/(1-specificity).

Negative likelihood ratio: (1-sensitivity)/specificity.

Area under the ROC curve is defined as the area under the sensitivity vs. 1-specificity curve.

## Discussion

### Main results

In this study, a cohort of 141 patients scheduled for primary TKA were followed from the moment of their enrolment on the waiting list until 6 months after the surgery. The objective of the study was to develop a prediction tool that would allow the early identification of patients at risk of poor outcome following primary TKA.

Important determinants of TKA outcomes measured at enrolment on the pre-surgery wait list were considered in the process of building the prediction rule. While the choice of the final predictive model could have been made from several criteria, we decided that the rule demonstrating the best sensitivity and an acceptable level of specificity would be the most appropriate because such a tool could identify patients at risk with fewer false negatives. Consequently, a model with a sensitivity of 82.1% and a specificity of 71.7% was chosen. Compared to published standards in clinical epidemiology, the model presented a somewhat weak positive likelihood ratio of 2.90 (95% CI 2.06 to 4.08) [39]. Nevertheless, this rule presented the best overall predictive validity and is comparable to other PR found to be valid in the literature. For example, the positive likelihood ratio of the final PR is higher than the value reported for the Ottawa Knee Rule (2.18, 95% CI 2.04 to 2.33) designed to identify the necessity for use of radiography in the emergency room in cases of acute knee injuries [25] or for the 5-item Cassandra rule allowing the identification of patients at risk of long-term back-related functional limitations (1.95, 95% CI 1.75 to 2.17) [26].

To our knowledge, no such tool has ever been built for candidates waiting for TKA or any other type of total joint replacement. A priority-setting tool for TKA and total hip arthroplasty has been developed and validated by the Western Canada Waiting List project partnership [40, 41]. This tooI allows for the quantification of the level of urgency of the status of patients enrolled on a waiting list for total knee or hip replacement, but its predictive capabilities have not been investigated.

The best predictive model developed in this study incorporates 5 items from the baseline WOMAC questionnaire, specifically questions regarding baseline function and stiffness, although an extensive set of known determinants were considered as potential predictors. The fact that four questions are related to pre-operative function is consistent with the literature where pre-operative function is a major determinant of post-operative function [12]. It is noteworthy to mention however that the current model does not include items regarding the level of pain. This can be due to the fact that patients undergoing TKA generally experience a notable relief in their pain level following the surgery but may still experience important disabilities [4–7]. It must be remembered, however, that the statistical approach used in building the PR does not allow for the interpretation of relationships as causal [42].

When building the PR, we intended to develop an applicable tool. Orthopedic surgeons and staff who assess the patients’ status upon placing them on TKA wait lists could be the main users of the PR. However, the PR could eventually also be used by other healthcare professionals, namely physician assistants, physiotherapists, occupational therapists, and nurses. In this way, any healthcare professional who takes charge of the case can take into account the results of the PR when determining the most appropriate course of action for the patient’s care. The PR shows promising practical implications, as it is relatively simple and easy to use in a clinical setting.

At times, the interpretation process may seem counterintuitive. Question 2 of the rule indicates that a patient may be categorized at risk if they experience mild difficulty when getting on or off the toilet, yet, depending on their answer to the Question 4, they may be classified as not at risk if they encounter severe difficulty performing the same task. Since the PR includes four items from the function subscale of the WOMAC, it would be expected that it classifies at risk those patients whose pre-operative function is severely affected, considering that pre-operative function has been consistently identified as a determinant of functional outcome [12]. Nevertheless, a predictor is not necessarily a determinant; its purpose is to predict the desired outcome and the development with recursive partitioning determined that it is this answer pattern that shows the best predictive value.

### Strengths of the study

This study followed a prospective longitudinal cohort design. It had high participation and follow-up proportions. There does not seem to be a selection bias, as there were no significant differences between participants and eligible non participants on age and gender as well as no significant differences in terms of pain, functional limitations at enrolment on the pre-surgery wait lists between subjects lost to follow-up, subjects who did not undergo surgery and participants who completed the interview six months after surgery (data not shown). Baseline measurements of the dependent variables were made as soon as the patients were enrolled on the pre-surgery wait list (mean ± SD: 12.6 ± 4.7 days).

### Limitations of the study

The sample of 141 patients that was used to derive the PR was small. This may diminish the applicability of the PR to the general population. Moreover, the population under study was patients undergoing primary TKA. This effectively may disregard patients with revision or bilateral TKA. Furthermore, the clinical outcomes of TKA were assessed using the WOMAC questionnaire, a self-reported measure. Performance-based measures such as the Timed Up and Go Test or the Six Minute Walking Test could have been used in order to complement the information recorded by the self-reported measure [43]. An assessment of the patients’ status in a more comprehensive manner could have thusly been achieved. In addition, we considered the patients having postoperative WOMAC scores in the first quintile to have a poor outcome. Since there is no consensus on what constitutes an appropriate measure of poor outcome following TKA, we decided that this method could be applied. It is important to point out that this PR allows for the identification of patients at risk of poor outcomes in the short-term following TKA and was not tested to predict long-term outcomes. The time point of six months after surgery was chosen to identify patients with poor outcomes, as it is a critical time in the patients’ rehabilitation period when they are often seen by surgeons to monitor progress and where the rehabilitation protocol and conservative treatment options may be easily modified if recovery is not optimal. Finally, the final PR has not yet been validated with a different sample of patients, its predictive validity has not been compared to the clinical judgment alone, and the clinical and financial impacts of its use have not been assessed yet. Until these further research steps are completed, the PR should be used with caution.

## Conclusion

The PR developed in the current study has the potential to identify patients at risk of poor surgical outcomes following TKA. Such patients could then be assigned to an appropriate course of action, such as prehabilitation, conservative management, wait list priority or intensive post-operative rehabilitation. These conducts may diminish the extent of deterioration of patients waiting for TKA and could decrease the socioeconomic burden of TKA. A further validation in an external cohort is needed. Impact analysis determining the usefulness of the rule in the clinical setting regarding cost-benefit, time and resource allocation as well as patient satisfaction is equally required.

## Electronic supplementary material

Additional file 1:**STROBE Statement—Checklist of items that should be included in reports of** ***cohort studies.***(DOC 87 KB)

Additional file 2:Appendix: contains eight prediction rules that were also considered, along with their respective two by two tables and their validity measures.(DOCX 722 KB)

Below are the links to the authors’ original submitted files for images.

Authors’ original file for figure 1

Authors’ original file for figure 2

Authors’ original file for figure 3

## Abbreviations

AUC ROC: Area under the receiver operating characteristics curve
CI: Confidence interval
OA: Osteoarthritis
PR: Prediction rule
PSI: Psychological symptom index
SD: Standard deviation
STROBE: Strengthening the reporting of observational studies in epidemiology
TKA: Total knee arthroplasty
WOMAC: Western Ontario and McMaster osteoarthritis index
