# Supplementary material for: Prediction of poor outcomes six months following total knee arthroplasty in patients awaiting surgery
Source: BMC Musculoskelet Disord. 2014 Sep 8;15:299. doi: 10.1186/1471-2474-15-299 (PMC4247215; doi:10.1186/1471-2474-15-299)
Supplement: Supplementary file 2 — Additional file 2:Appendix: contains eight prediction rules that were also considered, along with their respective two by two tables and their validity measures.(DOCX 722 KB) [file 12891_2014_2318_MOESM2_ESM.docx]

**APPENDIX**

**
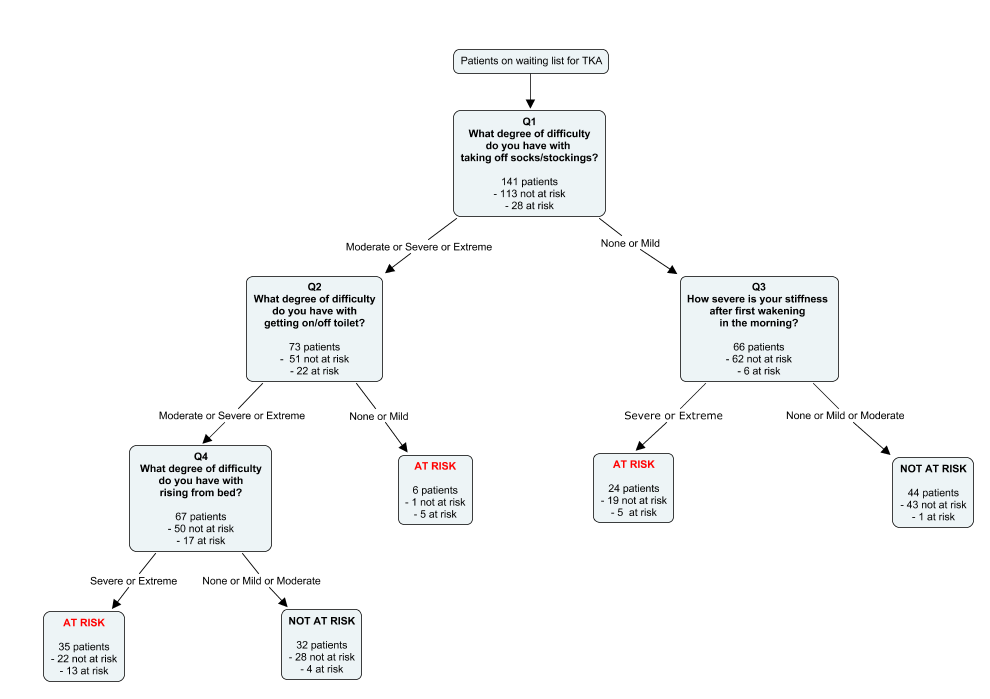
Figure 1** Graphical representation of Prediction Rule (PR) 1

**Table 1.1** Two by Two table of predicted versus actual outcomes of the PR1

|  | **Actual Outcome** | |
| --- | --- | --- |
| **Predicted outcome** | **AT RISK**  **(Post-operative WOMAC > 40.4)** | **NOT AT RISK**  **(Postoperative WOMAC ≤ 40.4)** |
| **AT RISK** | 23 | 42 |
| **NOT AT RISK** | 5 | 71 |
| *TOTAL* | *28* | *113* |

| **Measure** | **Estimates in training sample** |
| --- | --- |
| ***Sensitivity % (95% CI)*** | 82.1 (64.4-92.1) |
| ***Specificity % (95% CI)*** | 62.8 (53.6-71.2) |
| ***Positive predictive value % (95% CI)*** | 35.4 (24.9-47.5) |
| ***Negative predictive value % (95% CI)*** | 93.4 (85.5-97.2) |
| ***Positive likelihood ratio (95% CI)*** | 2.21 (1.65-2.97) |
| ***Negative likelihood ratio (95% CI)*** | 0.28 (0.13-0.63) |

**Table 1.2** Validity measures of the PR1

**Figure 2** Graphical representation of PR2

**
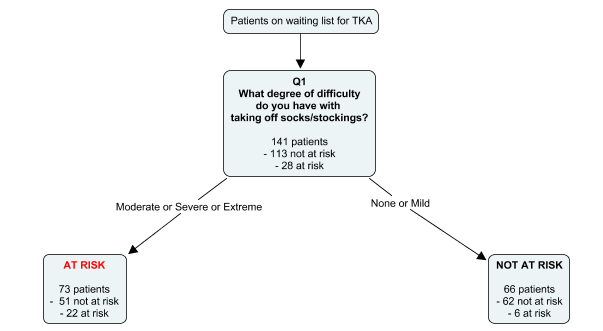
**

**Table 2.1** Two by Two table of predicted versus actual outcomes of the PR2

|  | **Actual Outcome** | |
| --- | --- | --- |
| **Predicted outcome** | **AT RISK**  **(Post-operative WOMAC > 40.4)** | **NOT AT RISK**  **(Postoperative WOMAC ≤ 40.4)** |
| **AT RISK** | 22 | 51 |
| **NOT AT RISK** | 6 | 62 |
| *TOTAL* | *28* | *113* |

**Table 2.2** Validity measures of the PR2

| **Measure** | **Estimates in training sample** |
| --- | --- |
| ***Sensitivity % (95% CI)*** | 78.6 (60.5-89.8) |
| ***Specificity % (95% CI)*** | 54.9 (45.7-63.7) |
| ***Positive predictive value % (95% CI)*** | 30.1 (20.8-41.4) |
| ***Negative predictive value % (95% CI)*** | 91.2 (82.1-95.9) |
| ***Positive likelihood ratio (95% CI)*** | 1.74 (1.32-2.31) |
| ***Negative likelihood ratio (95% CI)*** | 0.39 (0.19-0.81) |

**Figure 3** Graphical representation of PR3

**
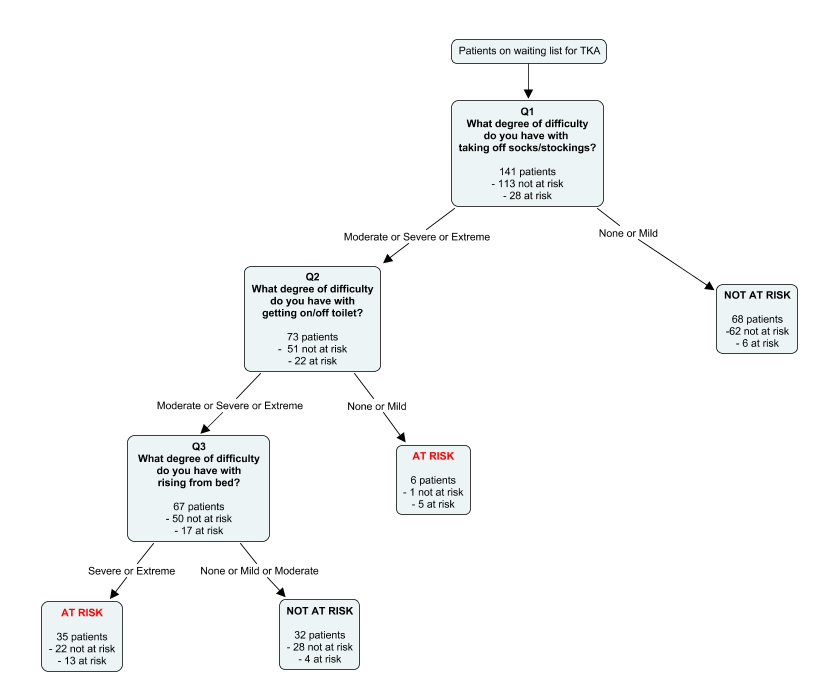
**

**Table 3.1** Two by Two table of predicted versus actual outcomes of the PR3

**Table 3.2** Validity measures of the PR3

| **Measure** | **Estimates in training sample** |
| --- | --- |
| ***Sensitivity % (95% CI)*** | 64.3 (45.8-79.3) |
| ***Specificity % (95% CI)*** | 79.6 (71.3-86.0) |
| ***Positive predictive value % (95% CI)*** | 43.9 (29.9-59.0) |
| ***Negative predictive value % (95% CI)*** | 90.0 (82.6-94.5) |
| ***Positive likelihood ratio (95% CI)*** | 3.16 (2.00-4.99) |
| ***Negative likelihood ratio (95% CI)*** | 0.39 (0.19-0.81) |

|  | **Actual Outcome** | |
| --- | --- | --- |
| **Predicted outcome** | **AT RISK**  **(Post-operative WOMAC > 40.4)** | **NOT AT RISK**  **(Postoperative WOMAC ≤ 40.4)** |
| **AT RISK** | 18 | 23 |
| **NOT AT RISK** | 10 | 90 |
| *TOTAL* | *28* | *113* |

**Figure 4** Graphical representation of PR4

**
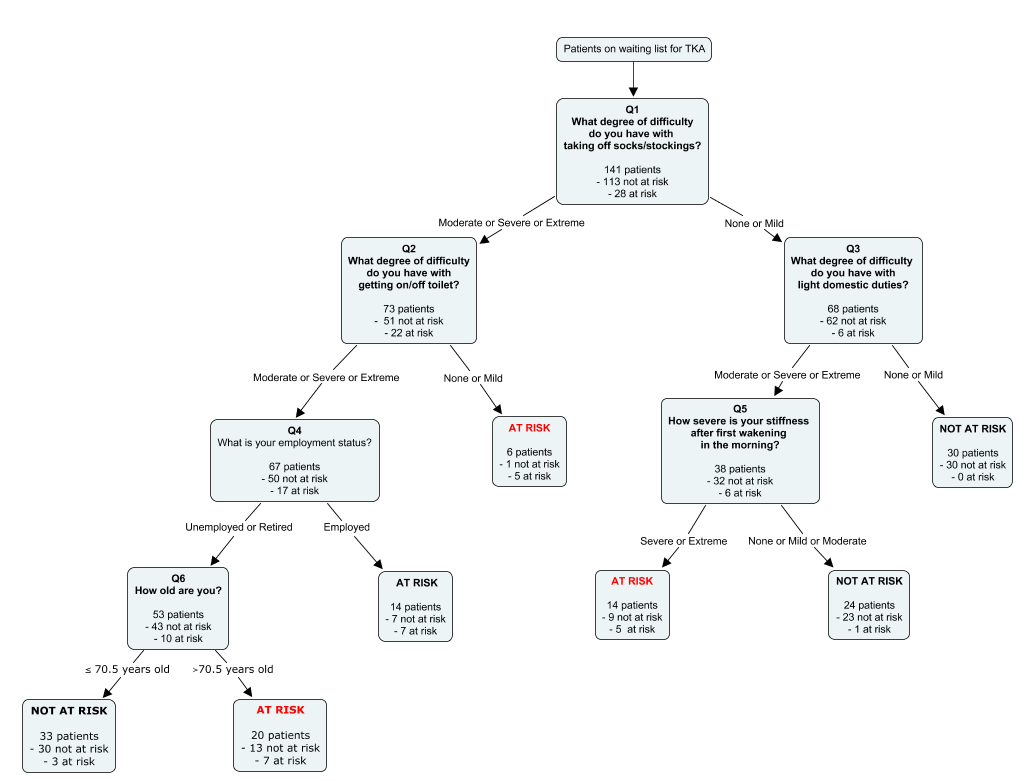
**

**Table 4.1** Two by Two table of predicted versus actual outcomes of the PR4

**Table 4.2** Validity measures of the PR4

| **Measure** | **Estimates in training sample** |
| --- | --- |
| ***Sensitivity % (95% CI)*** | 85.7 (68.5-94.3) |
| ***Specificity % (95% CI)*** | 73.5 (64.6-80.7) |
| ***Positive predictive value % (95% CI)*** | 44.4 (32.0-57.6) |
| ***Negative predictive value % (95% CI)*** | 95.4 (88.8-98.2) |
| ***Positive likelihood ratio (95% CI)*** | 3.23 (2.29-4.55) |
| ***Negative likelihood ratio (95% CI)*** | 0.19 (0.08-0.49) |

|  | **Actual Outcome** | |
| --- | --- | --- |
| **Predicted outcome** | **AT RISK**  **(Post-operative WOMAC > 40.4)** | **NOT AT RISK**  **(Postoperative WOMAC ≤ 40.4)** |
| **AT RISK** | 24 | 30 |
| **NOT AT RISK** | 4 | 83 |
| *TOTAL* | *28* | *113* |

**Figure 5** Graphical representation of PR5

**
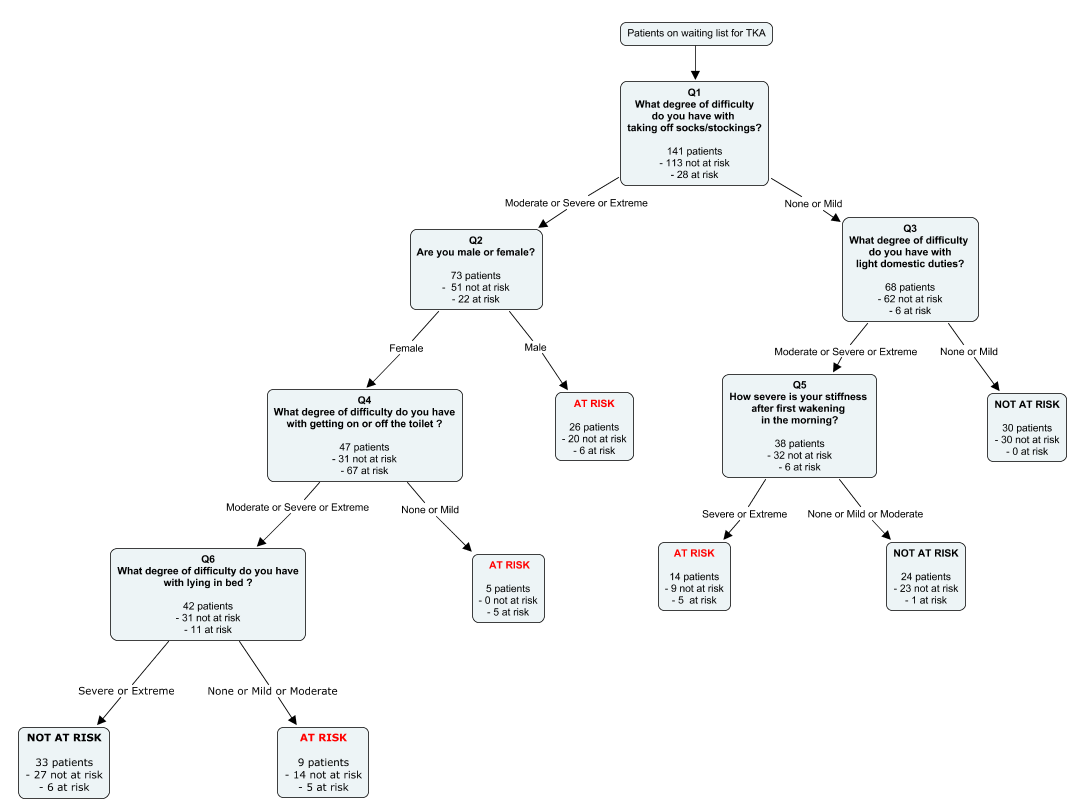
**

**Table 5.1** Two by Two table of predicted versus actual outcomes of the PR5

|  | **Actual Outcome** | |
| --- | --- | --- |
| **Predicted outcome** | **AT RISK**  **(Post-operative WOMAC > 40.4)** | **NOT AT RISK**  **(Postoperative WOMAC ≤ 40.4)** |
| **AT RISK** | 21 | 33 |
| **NOT AT RISK** | 7 | 80 |
| *TOTAL* | *28* | *113* |

| **Measure** | **Estimates in training sample** |
| --- | --- |
| ***Sensitivity % (95% CI)*** | 75.0 (56.6-87.3) |
| ***Specificity % (95% CI)*** | 70.8 (61.8-78.4) |
| ***Positive predictive value % (95% CI)*** | 38.9 (27.0-52.2) |
| ***Negative predictive value % (95% CI)*** | 92.0 (84.3-96.0) |
| ***Positive likelihood ratio (95% CI)*** | 2.57 (1.80-3.67) |
| ***Negative likelihood ratio (95% CI)*** | 0.35 (0.18-0.68) |

**Table 5.2** Validity measures of the PR5

**Figure 6** Graphical representation of PR6


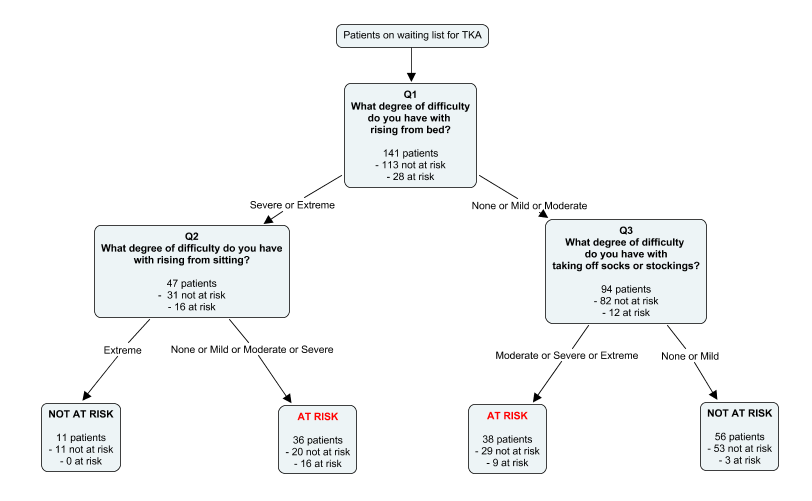


**Table 6.1** Two by Two table of predicted versus actual outcomes of the PR6

|  | **Actual Outcome** | |
| --- | --- | --- |
| **Predicted outcome** | **AT RISK**  **(Post-operative WOMAC > 40.4)** | **NOT AT RISK**  **(Postoperative WOMAC ≤ 40.4)** |
| **AT RISK** | 25 | 40 |
| **NOT AT RISK** | 3 | 73 |
| *TOTAL* | *28* | *113* |

**Table 6.2** Validity measures of the PR6

| **Measure** | **Estimates in training sample** |
| --- | --- |
| ***Sensitivity % (95% CI)*** | 89.3 (72.8-96.3) |
| ***Specificity % (95% CI)*** | 64.6 (55.4-72.8) |
| ***Positive predictive value % (95% CI)*** | 38.5 (27.6-50.6) |
| ***Negative predictive value % (95% CI)*** | 96.1 (89.0-98.6) |
| ***Positive likelihood ratio (95% CI)*** | 2.52 (1.91-3.34) |
| ***Negative likelihood ratio (95% CI)*** | 0.17 (0.06-0.49) |


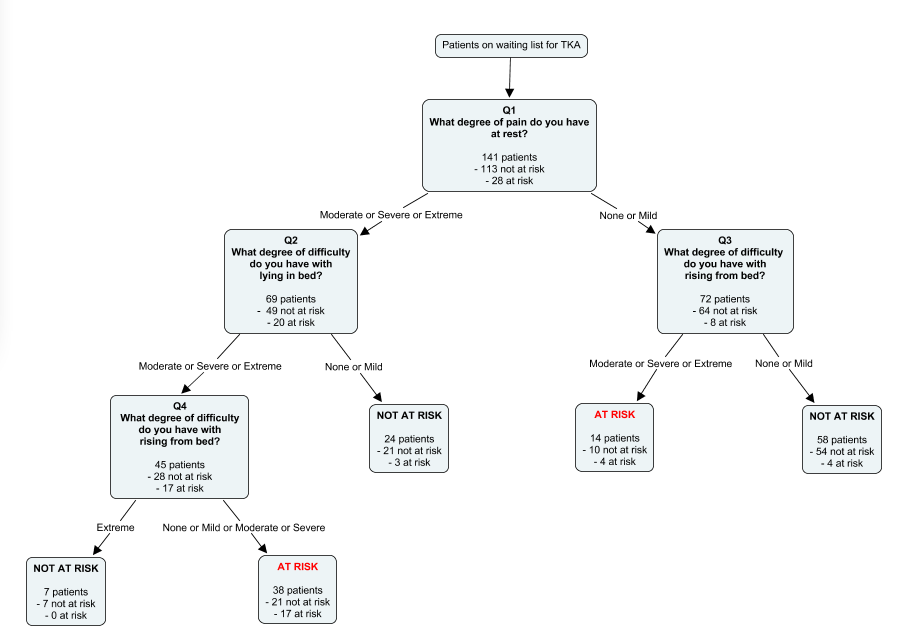
**Figure 7** Graphical representation of PR7

**Table 7.1** Two by Two table of predicted versus actual outcomes of the PR7

|  | **Actual Outcome** | |
| --- | --- | --- |
| **Predicted outcome** | **AT RISK**  **(Post-operative WOMAC > 40.4)** | **NOT AT RISK**  **(Postoperative WOMAC ≤ 40.4)** |
| **AT RISK** | 21 | 31 |
| **NOT AT RISK** | 7 | 82 |
| *TOTAL* | *28* | *113* |

**Table 7.2** Validity measures of the PR7

| **Measure** | **Estimates in training sample** |
| --- | --- |
| ***Sensitivity % (95% CI)*** | 89.3 (72.8-96.3) |
| ***Specificity % (95% CI)*** | 64.6 (55.4-72.8) |
| ***Positive predictive value % (95% CI)*** | 38.5 (27.6-50.6) |
| ***Negative predictive value % (95% CI)*** | 96.1 (89.0-98.6) |
| ***Positive likelihood ratio (95% CI)*** | 2.52 (1.91-3.34) |
| ***Negative likelihood ratio (95% CI)*** | 0.17 (0.06-0.49) |

**Figure 8** Graphical representation of PR8


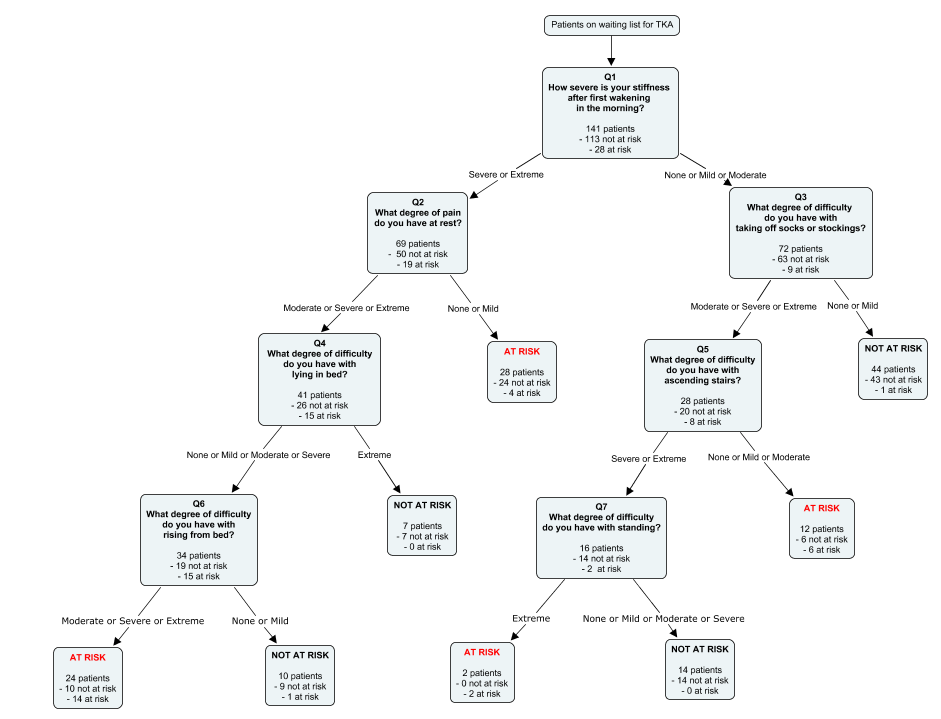


**Table 8.1** Two by Two table of predicted versus actual outcomes of the PR8

|  | **Actual Outcome** | |
| --- | --- | --- |
| **Predicted outcome** | **AT RISK**  **(Post-operative WOMAC > 40.4)** | **NOT AT RISK**  **(Postoperative WOMAC ≤ 40.4)** |
| **AT RISK** | 26 | 40 |
| **NOT AT RISK** | 2 | 73 |
| *TOTAL* | *28* | *113* |

**Table 8.2** Validity measures of the PR8

| **Measure** | **Estimates in training sample** |
| --- | --- |
| ***Sensitivity % (95% CI)*** | 92.9 (77.4-98.0) |
| ***Specificity % (95% CI)*** | 64.6 (55.4-72.8) |
| ***Positive predictive value % (95% CI)*** | 39.4 (28.5-51.5) |
| ***Negative predictive value % (95% CI)*** | 97.3 (90.8-99.3) |
| ***Positive likelihood ratio (95% CI)*** | 2.62 (2.00-3.43) |
| ***Negative likelihood ratio (95% CI)*** | 0.11 (0.03-0.42) |
