# Supplementary figures and images for: Prediction of poor outcomes six months following total knee arthroplasty in patients awaiting surgery
Source: BMC Musculoskelet Disord. 2014 Sep 8;15:299. doi: 10.1186/1471-2474-15-299 (PMC4247215; doi:10.1186/1471-2474-15-299)

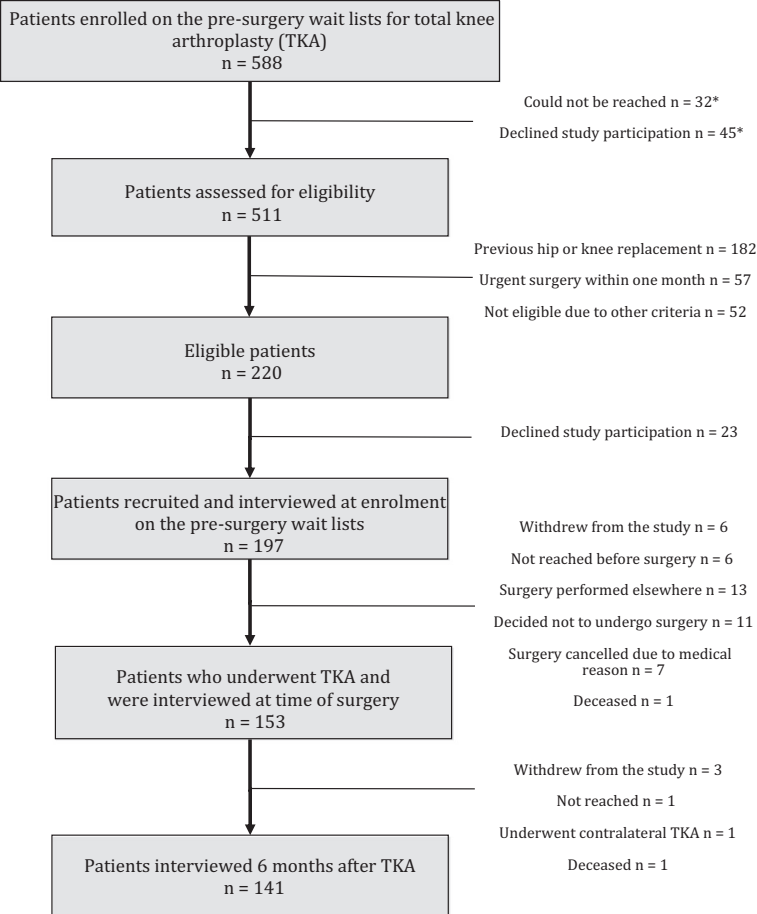

Supplement: Supplementary file 3 — Authors’ original file for figure 1 [file 12891_2014_2318_MOESM3_ESM.pdf]

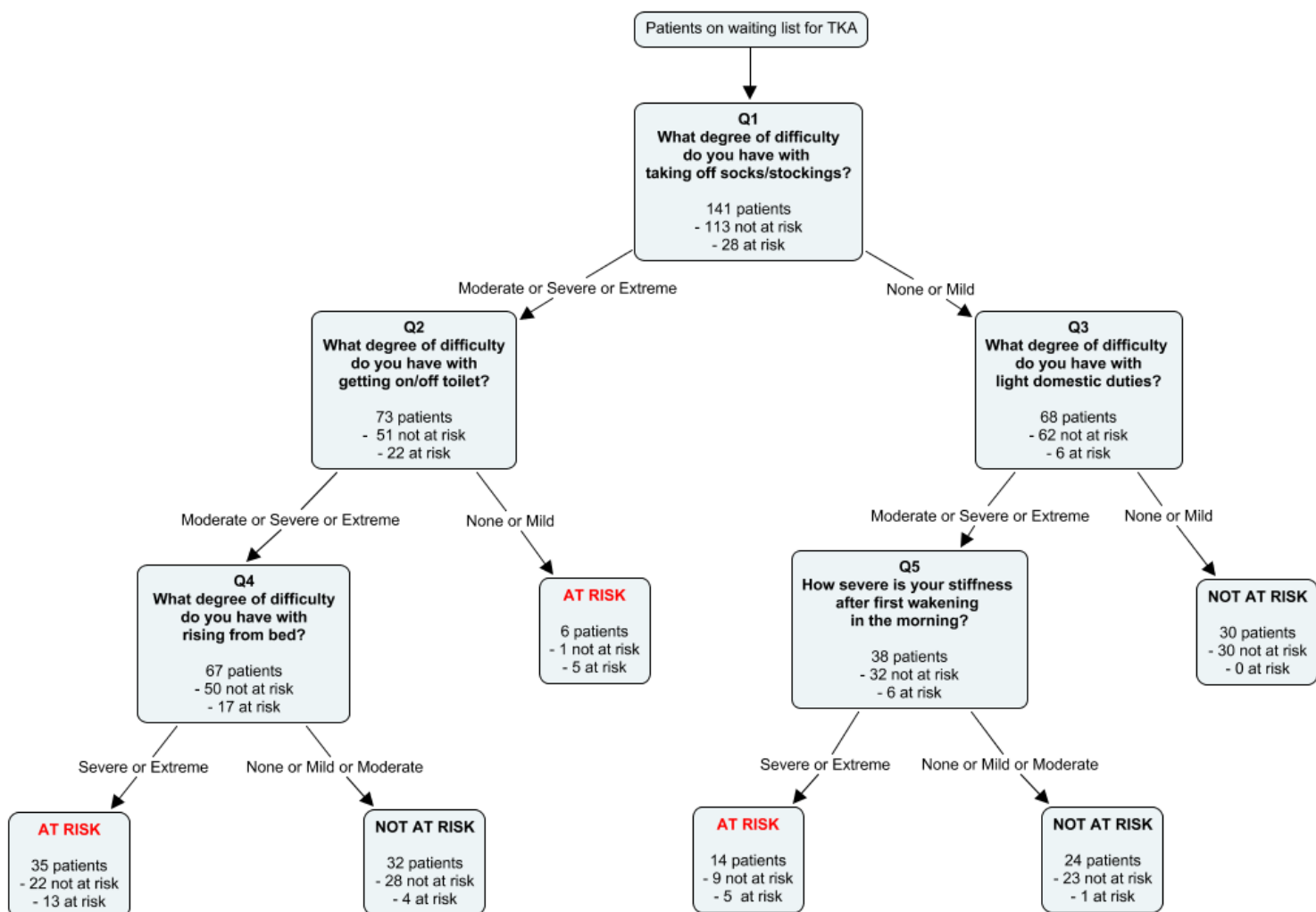

Supplement: Supplementary file 5 — Authors’ original file for figure 3 [file 12891_2014_2318_MOESM5_ESM.pdf]
