# Supplementary material for: Prediction of poor outcomes six months following total knee arthroplasty in patients awaiting surgery
Source: BMC Musculoskelet Disord. 2014 Sep 8;15:299. doi: 10.1186/1471-2474-15-299 (PMC4247215; doi:10.1186/1471-2474-15-299)
Supplement: Supplementary file 4 — Authors’ original file for figure 2 [file 12891_2014_2318_MOESM4_ESM.pdf]

INSTRUCTIONS: This survey asks for your view about your knee. This information will help us keep track of how you feel about your knee and how well you are able to do your usual activities.

Answer every question by checking the appropriate box. If you are unsure about how to answer a question, please give the best answer you can.

The following questions concern your physical function. By this we mean your ability to move around and to look after yourself. For each of the following activities, please indicate the degree of difficulty you have experienced in the **last 48 hours** due to your knee.

1. Taking off socks/stockings

None	Mild	Moderate	Severe	Extreme
▼	▼	▼	▼	▼
☐1	☐2	☐3	☐4	☐5

2. Getting on the toilet

None	Mild	Moderate	Severe	Extreme
▼	▼	▼	▼	▼
☐1	☐2	☐3	☐4	☐5

3. Light domestic duties

None	Mild	Moderate	Severe	Extreme
▼	▼	▼	▼	▼
☐1	☐2	☐3	☐4	☐5

4. Rising from bed

None	Mild	Moderate	Severe	Extreme
▼	▼	▼	▼	▼
☐1	☐2	☐3	☐4	☐5

The following question concerns the amount of joint stiffness you have experienced during the **last 48 hours** in your knee. Stiffness is a sensation of restriction or slowness in the ease with which you move your knee joint.

5. How severe is your knee joint stiffness after first awakening in the morning?

None	Mild	Moderate	Severe	Extreme
▼	▼	▼	▼	▼
☐1	☐2	☐3	☐4	☐5

INTERPRETATION:

The patient is **AT RISK** in case of any of the following combination of answers:

Q1: Moderate (3) or Severe (4) or Extreme (5)
Q2: Moderate (3) or Severe (4) or Extreme (5)
Q4: Severe (4) Extreme (5)

OR

Q1: Moderate (3) or Severe (4) or Extreme (5)
Q2: None (1) or Mild (2)

OR

Q1: None (1) or Mild (2)
Q3: Moderate (3) or Severe (4) or Extreme (5)
Q5: Severe (4) or Extreme (5)
